# Supplementary material for: Diagnostic accuracy of serological tests for the diagnosis of Chikungunya virus infection: A systematic review and meta-analysis
Source: PLoS Negl Trop Dis. 2022 Feb 4;16(2):e0010152. doi: 10.1371/journal.pntd.0010152 (PMC8849447; doi:10.1371/journal.pntd.0010152)
Supplement: S2 Checklist — (DOCX) [file pntd.0010152.s002.docx]

**S2** **Checklist** PRISMA DTA for abstract

| **Section/topic** | **#** | **PRISMA-DTA for Abstracts Checklist item** | **Abstract section #** |
| --- | --- | --- | --- |
| **TITLE and PURPOSE** | | |  |
| Title | 1 | Identify the report as a systematic review (+/- meta-analysis) of diagnostic test accuracy (DTA) studies. | Title |
| Objectives | 2 | Indicate the research question, including components such as participants, index test, and target conditions. | Background |
| **METHODS** | | |  |
| Eligibility criteria | 3 | Include study characteristics used as criteria for eligibility. | Methodology and principal findings  # First paragraph |
| Information sources | 4 | List the key databases searched and the search dates. | Methodology and principal findings  # First paragraph |
| Risk of bias & applicability | 5 | Indicate the methods of assessing risk of bias and applicability. | Methodology and principal findings  # First paragraph |
| Synthesis of results | A1 | Indicate the methods for the data synthesis. | Methodology and principal findings  # First paragraph |
| **RESULTS** | | |  |
| Included studies | 6 | Indicate the number and type of included studies and the participants and relevant characteristics of the studies (including the reference standard). | Methodology and principal findings  # Second paragraph |
| Synthesis of results | 7 | Include the results for the analysis of diagnostic accuracy, preferably indicating the number of studies and participants. Describe test accuracy including variability; if meta-analysis was done, include summary results and confidence intervals. | Methodology and principal findings  # Second paragraph |
| **DISCUSSION** | | |  |
| Strengths and limitations | 9 | Provide a brief summary of the strengths and limitations of the evidence | Methodology and principal findings  # Second paragraph |
| Interpretation | 10 | Provide a general interpretation of the results and the important implications. | Conclusion |
| **OTHER** | | |  |
| Funding | 11 | Indicate the primary source of funding for the review. | NA |
| Registration | 12 | Provide the registration number and the registry name | Registration |
