# Supplementary material for: Diagnostic accuracy of serological tests for the diagnosis of Chikungunya virus infection: A systematic review and meta-analysis
Source: PLoS Negl Trop Dis. 2022 Feb 4;16(2):e0010152. doi: 10.1371/journal.pntd.0010152 (PMC8849447; doi:10.1371/journal.pntd.0010152)
Supplement: S1 Appendix — (DOCX) [file pntd.0010152.s003.docx]

**S1 Appendix**

Search strategy (PubMed and CINAHL Complete)

1. Chikungunya [Title]
2. Test* [Title/Abstract] OR diagnos* [Title/Abstract] OR assay* [Title/Abstract] OR serolog* [Title/Abstract]
3. Sensitivity [Text Word]
4. #1 AND #2 AND #3

Search strategy (Scopus)

1. Chikungunya [Title]
2. Test* [Title-Abstract-Keyword] OR diagnos* [Title-Abstract-Keyword] OR assay* [Title-Abstract-Keyword] OR serolog* [Title-Abstract-Keyword]
3. Sensitivity [All fields]
4. #1 AND #2 AND #3
