# Supplementary material for: Diagnostic accuracy of serological tests for the diagnosis of Chikungunya virus infection: A systematic review and meta-analysis
Source: PLoS Negl Trop Dis. 2022 Feb 4;16(2):e0010152. doi: 10.1371/journal.pntd.0010152 (PMC8849447; doi:10.1371/journal.pntd.0010152)
Supplement: S2 Appendix — (DOCX) [file pntd.0010152.s004.docx]

**S2 Appendix**

**QUADAS-2 Validation form**

A systematic review with meta-analysis on the diagnostic accuracy of serological tests for chikungunya

| **Journal Article (Include author, title, reference, year of publication)** | | | | | | | | |
| --- | --- | --- | --- | --- | --- | --- | --- | --- |
| **Checklist completed by:** | | | | | | | | |
| **Domain 1: Patient Selection** | | | | | | | | |
| ***Risk of bias*** | | | | | | | | |
| 1.1 | Was a consecutive or random sample of patients enrolled? | | Yes | No | | | Unclear | |
| 1.2 | Was a case-control design avoided? | | Yes | | No | | Unclear | |
| Could selection of patients have introduced bias? | | Low | | | | High | Unclear | |
| ***Applicability*** | | | | | | | | |
| Is there concern that the included patients and setting do not match the review question? | | | Low | | | High | Unclear | |
| **Domain 2: Index test (s)** | | | | | | | | |
| ***Risk of bias*** | | | | | | | | |
| 2.1 | Were the index test results interpreted without knowledge of the results of the reference standard? | | Yes | | | No | | Unclear |
| 2.2 | If a threshold was used, was it pre-specified? | | Yes | | | No | | Unclear |
| 2.3 | Were the borderline or equivocal results excluded from analysis? | | Yes | | | No | | Unclear |
| Could the conduct or interpretation of the index test have introduced bias? | | Low | | | | High | Unclear | |
| ***Applicability*** | | | | | | | | |
| Is there concern that the index test, its conduct and its interpretation differ from the review question? | | | Low | | | High | | Unclear |
| **Domain 3: Reference standard** | | | | | | | | |
| ***Risk of bias*** | | | | | | | | |
| 3.1 | Were the reference standards likely to correctly classify the target condition? | | Yes | | | No | | Unclear |
| 3.2 | Were the reference standard results interpreted without knowledge of the results of the index tests? | | Yes | | | No | | Unclear |
| Could the reference standard, its conduct, or its interpretation have introduced bias? | | Low | | | | High | Unclear | |
| ***Applicability*** | | | | | | | | |
| Is there concern that the target condition as defined by the reference standard does not match the question? | | | Low | | | High | | Unclear |
| **Domain 4: Flow and Timing** | | | | | | | | |
| ***Risk of bias*** | | | | | | | | |
| 4.1 | Was there an appropriate interval between index test and reference standard? | | Yes | | | No | | Unclear |
| 4.2 | Were all patients included in the analysis? | | Yes | | | No | | Unclear |
| Could the patient flow have introduced bias? | | Low | | | | High | Unclear | |

Note:

Yes – indicate low risk of bias

No – indicate high risk of bias

Unclear – when insufficient data are reported

(More than 1 signalling question answered “no” or “unclear”, risk of bias is high)
