# Supplementary figures and images for: Diagnostic accuracy of serological tests for the diagnosis of Chikungunya virus infection: A systematic review and meta-analysis
Source: PLoS Negl Trop Dis. 2022 Feb 4;16(2):e0010152. doi: 10.1371/journal.pntd.0010152 (PMC8849447; doi:10.1371/journal.pntd.0010152)

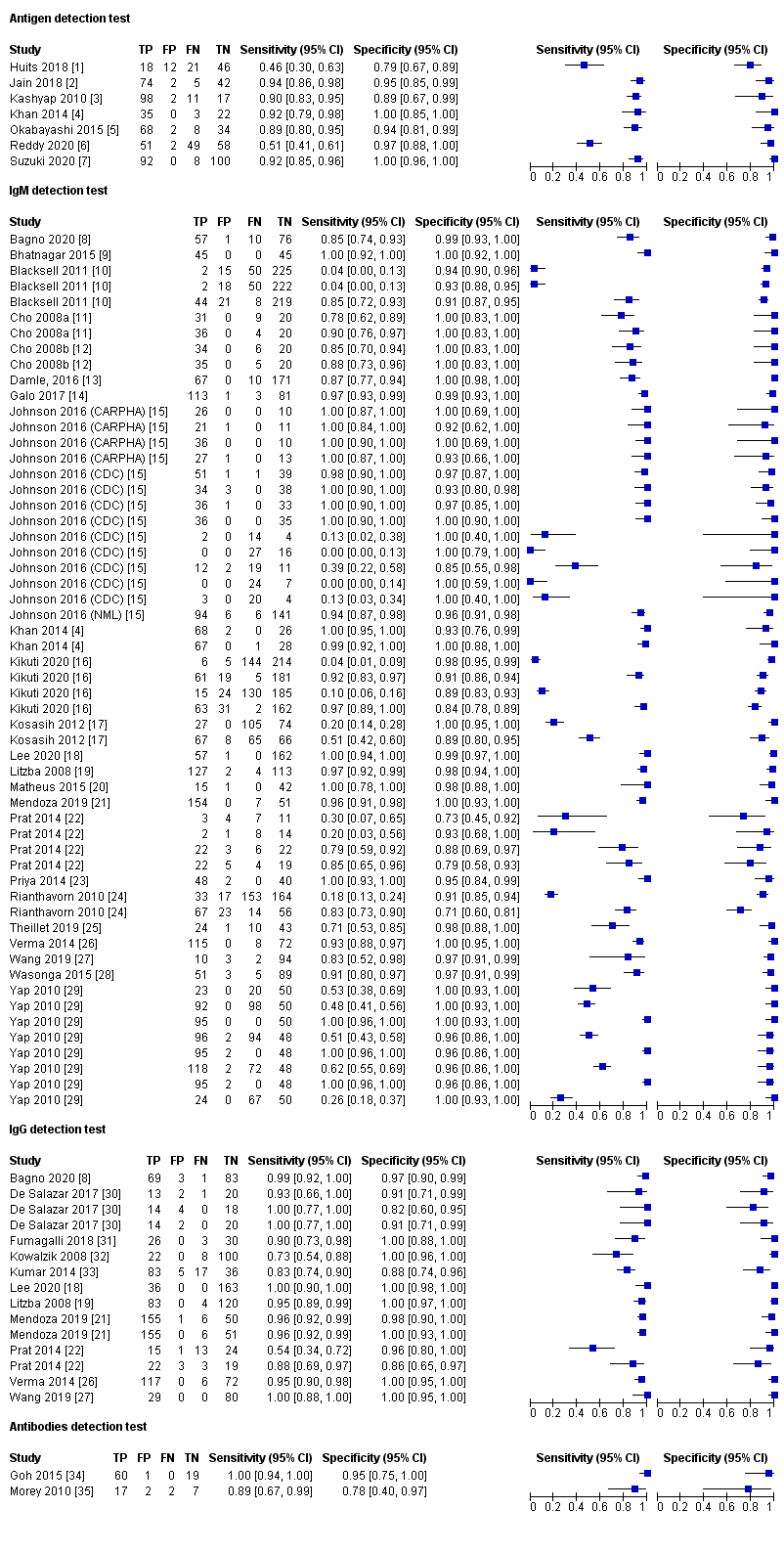

Supplement: S1 Fig — (TIF) [file pntd.0010152.s005.tif]

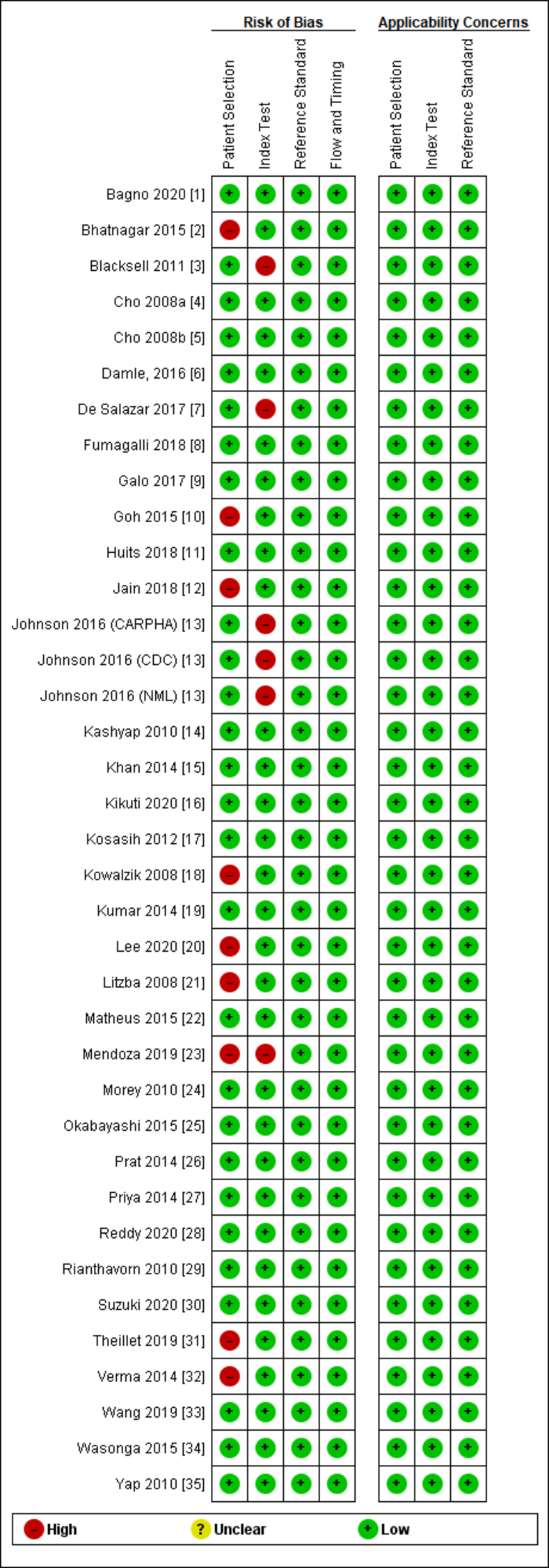

Supplement: S2 Fig — (TIF) [file pntd.0010152.s006.tif]
