# Supplementary material for: Diagnostic accuracy of serological tests for the diagnosis of Chikungunya virus infection: A systematic review and meta-analysis
Source: PLoS Negl Trop Dis. 2022 Feb 4;16(2):e0010152. doi: 10.1371/journal.pntd.0010152 (PMC8849447; doi:10.1371/journal.pntd.0010152)
Supplement: S1 Reference list — (PDF) [file pntd.0010152.s007.pdf]

Reference list for S1_Fig

1. Huits R, Okabayashi T, Cnops L, Barbé B, Van Den Berg R, Bartholomeeusen K, et al. Diagnostic accuracy of a rapid E1-antigen test for chikungunya virus infection in a reference setting. Clin Microbiol Infect. 2018;24(1):78-81.
2. Jain J, Okabayashi T, Kaur N, Nakayama E, Shioda T, Gaind R, et al. Evaluation of an immunochromatography rapid diagnosis kit for detection of chikungunya virus antigen in India, a dengue-endemic country. Virol J. 2018;15(1):84.
3. Kashyap RS, Morey SH, Ramteke SS, Chandak NH, Parida M, Deshpande PS, et al. Diagnosis of Chikungunya fever in an Indian population by an indirect enzyme-linked immunosorbent assay protocol based on an antigen detection assay: a prospective cohort study. Clin Vaccine Immunol. 2010;17(2):291-7.
4. Khan M, Dhanwani R, Kumar JS, Rao PV, Parida M. Comparative evaluation of the diagnostic potential of recombinant envelope proteins and native cell culture purified viral antigens of Chikungunya virus. J Med Virol. 2014;86(7):1169-75.
5. Okabayashi T, Sasaki T, Masrinoul P, Chantawat N, Yoksan S, Nitatpattana N, et al. Detection of chikungunya virus antigen by a novel rapid immunochromatographic test. J Clin Microbiol. 2015;53(2):382-8.
6. Reddy A, Bosch I, Salcedo N, Herrera BB, de Puig H, Narváez CF, et al. Development and Validation of a Rapid Lateral Flow E1/E2-Antigen Test and ELISA in Patients Infected with Emerging Asian Strain of Chikungunya Virus in the Americas. Viruses. 2020;12(9).
7. Suzuki K, Huits R, Phadungsombat J, Tuekprakhon A, Nakayama EE, van den Berg R, et al. Promising application of monoclonal antibody against chikungunya virus E1-antigen across genotypes in immunochromatographic rapid diagnostic tests. Virol J. 2020;17(1):90.
8. Bagno FF, Godói LC, Figueiredo MM, Rodrigues Sérgio SA, de Fátima Silva Moraes T, de Castro Salazar N, et al. Chikungunya E2 protein produced in E. coli and HEK293-T cells—comparison of their performances in ELISA. Viruses. 2020.
9. Bhatnagar S, Kumar P, Mohan T, Verma P, Parida MM, Hoti SL, et al. Evaluation of multiple antigenic peptides based on the Chikungunya E2 protein for improved serological diagnosis of infection. Viral Immunol. 2015;28(2):107-12.
10. Blacksell SD, Tanganuchitcharnchai A, Jarman RG, Gibbons RV, Paris DH, Bailey MS, et al. Poor diagnostic accuracy of commercial antibody-based assays for the diagnosis of acute Chikungunya infection. Clin Vaccine Immunol. 2011;18(10):1773-5.
11. Cho B, Jeon BY, Kim J, Noh J, Kim J, Park M, et al. Expression and evaluation of Chikungunya virus E1 and E2 envelope proteins for serodiagnosis of Chikungunya virus infection. Yonsei Med J. 2008;49(5):828-35.
12. Cho B, Kim J, Cho JE, Jeon BY, Park S. Expression of the capsid protein of Chikungunya virus in a baculovirus for serodiagnosis of Chikungunya disease. J Virol Methods. 2008;154(1-2):154-9.
13. Damle RG, Jayaram N, Kulkarni SM, Nigade K, Khutwad K, Gosavi S, et al. Diagnostic potential of monoclonal antibodies against the capsid protein of chikungunya virus for detection of recent infection. Arch Virol. 2016;161(6):1611-22.
14. Galo SS, González K, Téllez Y, García N, Pérez L, Gresh L, et al. Development of in-house serological methods for diagnosis and surveillance of chikungunya. Rev Panam Salud Publica. 2017;41:e56.
15. Johnson BW, Goodman CH, Holloway K, de Salazar PM, Valadere AM, Drebot MA. Evaluation of Commercially Available Chikungunya Virus Immunoglobulin M Detection Assays. Am J Trop Med Hyg. 2016;95(1):182-92.
16. Kikuti M, Tauro LB, Moreira PSS, Nascimento LCJ, Portilho MM, Soares GC, et al. Evaluation of two commercially available chikungunya virus IgM enzyme-linked immunoassays (ELISA) in a setting of concomitant transmission of chikungunya, dengue and Zika viruses. Int J Infect Dis. 2020;91:38-43.

17. Kosasih H, Widjaja S, Surya E, Hadiwijaya SH, Butarbutar DP, Jaya UA, et al. Evaluation of two IgM rapid immunochromatographic tests during circulation of Asian lineage Chikungunya virus. Southeast Asian J Trop Med Public Health. 2012;43(1):55-61.
18. Lee H, Ryu JH, Yun S, Jang JH, Choi AR, Cho SY, et al. Evaluation of a Newly Developed Rapid Automated Fluorescent Lateral Flow Immunoassay to Detect IgG and IgM Antibodies to Chikungunya Virus. Infect Chemother. 2020;52(4):611-5.
19. Litzba N, Schuffenecker I, Zeller H, Drosten C, Emmerich P, Charrel R, et al. Evaluation of the first commercial chikungunya virus indirect immunofluorescence test. J Virol Methods. 2008;149(1):175-9.
20. Matheus S, Huc P, Labeau B, Bremand L, Enfissi A, Merle O, et al. The use of serum spotted onto filter paper for diagnosing and monitoring Chikungunya virus infection. J Clin Virol. 2015;71:89-92.
21. Mendoza EJ, Robinson A, Dimitrova K, Mueller N, Holloway K, Makowski K, et al. Combining anti-IgM and IgG immunoassays for comprehensive chikungunya virus diagnostic testing. Zoonoses Public Health. 2019;66(8):909-17.
22. Prat CM, Flusin O, Panella A, Tenebray B, Lanciotti R, Leparc-Goffart I. Evaluation of commercially available serologic diagnostic tests for chikungunya virus. Emerg Infect Dis. 2014;20(12):2129-32.
23. Priya R, Khan M, Rao MK, Parida M. Cloning, expression and evaluation of diagnostic potential of recombinant capsid protein based IgM ELISA for chikungunya virus. J Virol Methods. 2014;203:15-22.
24. Rianthavorn P, Wuttirattanakowit N, Prianantathavorn K, Limpaphayom N, Theamboonlers A, Poovorawan Y. Evaluation of a rapid assay for detection of IgM antibodies to chikungunya. Southeast Asian J Trop Med Public Health. 2010;41(1):92-6.
25. Theillet G, Grard G, Galla M, Maisse C, Enguehard M, Cresson M, et al. Detection of chikungunya virus-specific IgM on laser-cut paper-based device using pseudo-particles as capture antigen. J Med Virol. 2019;91(6):899-910.
26. Verma P, Bhatnagar S, Kumar P, Chattree V, Parida MM, Hoti SL, et al. Analysis of antibody response (IgM, IgG, IgG3) to Chikungunya virus using a panel of peptides derived from envelope protein for serodiagnosis. Clinical Chemistry and Laboratory Medicine. 2014.
27. Wang R, Ongagna-Yhombi SY, Lu Z, Centeno-Tablante E, Colt S, Cao X, et al. Rapid Diagnostic Platform for Colorimetric Differential Detection of Dengue and Chikungunya Viral Infections. Anal Chem. 2019;91(8):5415-23.
28. Wasonga C, Inoue S, Kimotho J, Morita K, Ongus J, Sang R, et al. Development and Evaluation of an in-House IgM-Capture ELISA for the Detection of Chikungunya and Its Application to a Dengue Outbreak Situation in Kenya in 2013. Jpn J Infect Dis. 2015;68(5):410-4.
29. Yap G, Pok KY, Lai YL, Hapuarachchi HC, Chow A, Leo YS, et al. Evaluation of Chikungunya diagnostic assays: differences in sensitivity of serology assays in two independent outbreaks. PLoS Negl Trop Dis. 2010;4(7):e753.
30. De Salazar PM, Valadere AM, Goodman CH, Johnson BW. Evaluation of three commercially-available chikungunya virus immunoglobulin G immunoassays. Rev Panam Salud Publica. 2017;41:e62.
31. Fumagalli MJ, de Souza WM, Espósito DLA, Silva A, Romeiro MF, Martinez EZ, et al. Enzyme-linked immunosorbent assay using recombinant envelope protein 2 antigen for diagnosis of Chikungunya virus. Virol J. 2018;15(1):112.
32. Kowalzik S, Xuan NV, Weissbrich B, Scheiner B, Schied T, Drosten C, et al. Characterisation of a chikungunya virus from a German patient returning from Mauritius and development of a serological test. Medical Microbiology and Immunology. 2008;197(4):381-6.
33. Kumar P, Pok KY, Tan LK, Angela C, Leo YS, Ng LC. Development and evaluation of baculovirus-expressed Chikungunya virus E1 envelope proteins for serodiagnosis of Chikungunya infection. J Virol Methods. 2014;206:67-75.

34. Goh LYH, Kam YW, Metz SW, Hobson-Peters J, Prow NA, McCarthy S, et al. A sensitive epitope-blocking ELISA for the detection of Chikungunya virus-specific antibodies in patients. Journal of Virological Methods. 2015.
35. Morey SH, Kashyap RS, Purohit HJ, Taori GM, Daginawala HF. An approach towards peptide-based antibody detection for diagnosis of Chikungunya infection. Biomarkers. 2010;15(6):546-52.
