# Supplementary material for: Diagnostic accuracy of serological tests for the diagnosis of Chikungunya virus infection: A systematic review and meta-analysis
Source: PLoS Negl Trop Dis. 2022 Feb 4;16(2):e0010152. doi: 10.1371/journal.pntd.0010152 (PMC8849447; doi:10.1371/journal.pntd.0010152)
Supplement: S1 Table — Section A: Characteristics of commercial ELISA-based tests included in the meta-analysis. section B: Characteristics of commercial Immunofluorescence assays included in the meta-analysis. section C: Characteristics of commercial rapid tests included in the meta-analysis. (DOCX) [file pntd.0010152.s009.docx]

**S1 Table A** Characteristics of commercial ELISA-based tests included in the meta-analysis.

| **Author** | **Year** | **Study design** | | | **Reference test** | **Index test format** | | **Time of sample collection (day of post symptom onset)** | | **Total number of samples** | **TP** | | **FP** | | **FN** | | **TN** | | **Ref** | |
| --- | --- | --- | --- | --- | --- | --- | --- | --- | --- | --- | --- | --- | --- | --- | --- | --- | --- | --- | --- | --- |
| Anti-CHIKV ELISA (IgM), Euroimmun Lübeck, Germany | | | | | | | | | | | | | | | | | | | |  |
| Johnson (CDC) | 2016 | Partial cohort and case-control | | | CDC MAC-ELISA and PRNT | IgM Indirect ELISA | | 2 to 33 | | 92 | 51 | | 1 | | 1 | | 39 | | [1] | |
| Johnson (CARPHA) | 2016 | Partial cohort and case-control | | | CDC MAC-ELISA and PRNT | Indirect ELISA | | NA | | 36 | 26 | | 0 | | 0 | | 10 | | [1] | |
| Johnson (NML) | 2016 | Partial cohort and case-control | | | CDC MAC-ELISA and PRNT and/or qRT-PCR and/or hemagglutination inhibition assay | Indirect ELISA | | NA | | 247 | 94 | | 6 | | 6 | | 141 | | [1] | |
| Kikuti | 2020 | Cohort | | | RT-PCR | Indirect ELISA | | 8 to >30 | | 258 | 63 | | 31 | | 2 | | 162 | | [2] | |
| Mendoza | 2019 | Case-control | | | Plaque reduction neutralization test (PRNT) and/or RT-PCR | IgM Indirect ELISA | | NA | | 212 | 154 | | 0 | | 7 | | 51 | | [3] | |
| Prat | 2014 | Partial cohort and case-control | | | In-house MAC-ELISA and PRNT | IgM Indirect ELISA | | NA | | 50 | 22 | | 5 | | 4 | | 19 | | [4] | |
| Anti-CHIKV ELISA (IgG), Euroimmun Lübeck, Germany | | | | | | | | | | | | | | | | | | | |  |
| De Salazar | 2017 | | Partial cohort and case-control | In-house ELISA (CDC, Atlanta, United States) | | IgG Indirect ELISA | 15 to 90 | | 36 | | 14 | 4 | | 0 | | 18 | | [5] | |  |
| Mendoza | 2019 | | Case-control | Plaque reduction neutralization test and/or RT-PCR | | IgG Indirect ELISA | NA | | 212 | | 155 | 1 | | 6 | | 50 | | [3] | |  |
| Prat | 2014 | | Partial cohort and case-control | In-house ELISA and PRNT | | IgG Indirect ELISA | NA | | 47 | | 22 | 3 | | 3 | | 19 | | [4] | |  |

| **Author** | **Year** | **Study design** | | **Reference test** | | | **Index test format** | | **Time of sample collection (day of post symptom onset)** | **Total number of samples** | **TP** | **FP** | **FN** | **TN** | **Ref** |
| --- | --- | --- | --- | --- | --- | --- | --- | --- | --- | --- | --- | --- | --- | --- | --- |
| SD Chikungunya IgM ELISA, Standard Diagnostics Inc., Yongin-si, Korea | | | | | | | | | | | | | | | |
| Blacksell | 2011 | Cohort | | Hemagglutination inhibition (HI) and/or IgM antibody capture ELISA and/or RT-PCR | | | MAC-ELISA | | 19 to 30 | 292 | 44 | 21 | 8 | 219 | [6] |
| Johnson (CDC) | 2016 | Partial cohort and case-control | | CDC MAC-ELISA and PRNT | | | MAC-ELISA | | 2 to 33 | 44 | 12 | 2 | 19 | 11 | [1] |
| Anti-Chikungunya Virus IgM Human ELISA Kit, UK | | | | | | | | | | | | | | | |
| Johnson (CDC) | 2016 | Partial cohort and case-control | | | CDC MAC-ELISA and PRNT | | MAC-ELISA | | 2 to 33 | 70 | 36 | 1 | 0 | 33 | [1] |
| Johnson (CARPHA) | 2016 | Partial cohort and case-control | | | CDC MAC-ELISA and PRNT | | MAC-ELISA | | NA | 46 | 36 | 0 | 0 | 10 | [1] |
| CHIKjj Detect MAC-ELISA, InBios, Seattle, WA, USA | | | | | | | | | | | | | | | |
| Johnson (CDC) | 2016 | | Partial cohort and case-control | | | CDC MAC-ELISA and PRNT | MAC-ELISA | 2 to 33 | | 71 | 36 | 0 | 0 | 35 | [1] |
| Johnson (CARPHA) | 2016 | | Partial cohort and case-control | | | CDC MAC-ELISA and PRNT | MAC-ELISA | NA | | 41 | 27 | 1 | 0 | 13 | [1] |
| Kikuti | 2020 | | Cohort | | | RT-PCR | MAC-ELISA | 8 to >30 | | 266 | 61 | 19 | 5 | 181 | [2] |

Note: TP, true positive; FP, false positive; FN, false negative; TN, true negative; Ref, reference; NA, not available

**S1 Table B** Characteristics of commercial Immunofluorescence assays included in the meta-analysis.

| **Author** | **Year** | **Study design** | **Reference test** | | **Index test format** | | **Time of sample collection (day of post symptom onset)** | **Total number of samples** | **TP** | **FP** | **FN** | **TN** | **Ref** |
| --- | --- | --- | --- | --- | --- | --- | --- | --- | --- | --- | --- | --- | --- |
| Anti-CHIKV IIFT (IgG) (Euroimmun Lübeck, Germany) | | | | | | | | | | | | | |
| De Salazar | 2017 | Partial cohort and case-control | | In-house ELISA (CDC, Atlanta, United States) | IFA | 15 to 90 | | 36 | 14 | 2 | 0 | 20 | [5] |
| Litzba | 2008 | Case-control | | Indirect IgG ELISA or In-house IIFT | IFA | NA | | 207 | 83 | 0 | 4 | 120 | [7] |
| Anti-CHIKV IIFT (IgM) (Euroimmun Lübeck, Germany) | | | | | | | | | | | | | |
| Johnson (CDC) | 2016 | Partial cohort and case-control | CDC MAC-ELISA and PRNT | | IFA | | 2 to 33 | 75 | 34 | 3 | 0 | 38 | [1] |
| Johnson (CARPHA) | 2016 | Partial cohort and case-control | CDC MAC-ELISA and PRNT | | IFA | | NA | 33 | 21 | 1 | 0 | 11 | [1] |
| Litzba | 2008 | Case-control | In-house IgM capture ELISA or in-house IIFT | | IFA | | NA | 246 | 127 | 2 | 4 | 113 | [7] |
| Yap | 2010 | Partial cohort and case-control | RT-PCR and IgM serology | | IFA | | 7 to 40 | 145 | 95 | 0 | 0 | 50 | [8] |

Note: TP, true positive; FP, false positive; FN, false negative; TN, true negative; Ref, reference; NA, not available

**S1 Table C** Characteristics of commercial rapid tests included in the meta-analysis.

| **Author** | **Year** | **Study design** | **Reference test** | **Index test format** | **Time of sample collection (day of post symptom onset)** | **Total number of samples** | **TP** | **FP** | **FN** | **TN** | **Ref** |
| --- | --- | --- | --- | --- | --- | --- | --- | --- | --- | --- | --- |
| On-site CHIK IgM Combo Rapid test (CTK Biotech Inc., San Diego, CA, USA) | | | | | | | | | | | |
| Johnson (CDC) | 2016 | Partial cohort and case-control | CDC MAC-ELISA and PRNT | Rapid test | 2 to 33 | 27 | 3 | 0 | 20 | 4 | [1] |
| Prat | 2014 | Partial cohort and case-control | In-house MAC-ELISA and PRNT | Rapid test | NA | 25 | 2 | 1 | 8 | 14 | [4] |
| Yap | 2010 | Partial cohort and case-control | RT-PCR and IgM serology | Rapid test | 7 to 40 | 93 | 23 | 0 | 20 | 50 | [8] |
| SD BIOLINE Chikungunya IgM (Standard Diagnostics Inc., Yongin-si, Korea) | | | | | | | | | | | |
| Johnson (CDC) | 2016 | Partial cohort and case-control | CDC MAC-ELISA and PRNT | Rapid test | 2 to 33 | 31 | 0 | 0 | 24 | 7 | [1] |
| Prat | 2014 | Partial cohort and case-control | In-house MAC-ELISA and PRNT | Rapid test | NA | 25 | 3 | 4 | 7 | 11 | [4] |
| Rianthavorn | 2010 | Cohort | Semi-nested RT-PCR and ELISA kit (SD BIOLINE) | Rapid test | 7 to >14 | 160 | 67 | 23 | 14 | 56 | [9] |

Note: TP, true positive; FP, false positive; FN, false negative; TN, true negative; Ref, reference; NA, not available

References

1. Johnson BW, Goodman CH, Holloway K, de Salazar PM, Valadere AM, Drebot MA. Evaluation of Commercially Available Chikungunya Virus Immunoglobulin M Detection Assays. Am J Trop Med Hyg. 2016;95(1):182-92.

2. Kikuti M, Tauro LB, Moreira PSS, Nascimento LCJ, Portilho MM, Soares GC, et al. Evaluation of two commercially available chikungunya virus IgM enzyme-linked immunoassays (ELISA) in a setting of concomitant transmission of chikungunya, dengue and Zika viruses. Int J Infect Dis. 2020;91:38-43.

3. Mendoza EJ, Robinson A, Dimitrova K, Mueller N, Holloway K, Makowski K, et al. Combining anti-IgM and IgG immunoassays for comprehensive chikungunya virus diagnostic testing. Zoonoses Public Health. 2019;66(8):909-17.

4. Prat CM, Flusin O, Panella A, Tenebray B, Lanciotti R, Leparc-Goffart I. Evaluation of commercially available serologic diagnostic tests for chikungunya virus. Emerg Infect Dis. 2014;20(12):2129-32.

5. De Salazar PM, Valadere AM, Goodman CH, Johnson BW. Evaluation of three commercially-available chikungunya virus immunoglobulin G immunoassays. Rev Panam Salud Publica. 2017;41:e62.

6. Blacksell SD, Tanganuchitcharnchai A, Jarman RG, Gibbons RV, Paris DH, Bailey MS, et al. Poor diagnostic accuracy of commercial antibody-based assays for the diagnosis of acute Chikungunya infection. Clin Vaccine Immunol. 2011;18(10):1773-5.

7. Litzba N, Schuffenecker I, Zeller H, Drosten C, Emmerich P, Charrel R, et al. Evaluation of the first commercial chikungunya virus indirect immunofluorescence test. J Virol Methods. 2008;149(1):175-9.

8. Yap G, Pok KY, Lai YL, Hapuarachchi HC, Chow A, Leo YS, et al. Evaluation of Chikungunya diagnostic assays: differences in sensitivity of serology assays in two independent outbreaks. PLoS Negl Trop Dis. 2010;4(7):e753.

9. Rianthavorn P, Wuttirattanakowit N, Prianantathavorn K, Limpaphayom N, Theamboonlers A, Poovorawan Y. Evaluation of a rapid assay for detection of IgM antibodies to chikungunya. Southeast Asian J Trop Med Public Health. 2010;41(1):92-6.
