# Supplementary material for: Diagnostic accuracy of serological tests for the diagnosis of Chikungunya virus infection: A systematic review and meta-analysis
Source: PLoS Negl Trop Dis. 2022 Feb 4;16(2):e0010152. doi: 10.1371/journal.pntd.0010152 (PMC8849447; doi:10.1371/journal.pntd.0010152)
Supplement: S2 Table — (DOCX) [file pntd.0010152.s010.docx]

**S2 Table** Analysis of commercial vs in-house developed IgM tests with the exclusion of case-control study.

|  | Number of index test | Pooled Sensitivity | | *P*-value | Pooled Specificity | | *P*-value |
| --- | --- | --- | --- | --- | --- | --- | --- |
|  |  | Percentage  [95% CI] | *I*^2^  [95% CI] |  | Percentage  [95% CI] | *I*^2^  [95% CI] |  |
| Commercial vs In-house | | | | | | | |
| Commercial | 30 | 78.7  [49.1; 93.4] | 91.9%  [89.5; 93.7] | <0.001^a^ | 95.9  [93.1; 97.6] | 52.5%  [27.9; 68.7] | 0.041^a^ |
| In-house without case-control | 11 | 94.3  [85.3; 97.9] | 81.5%  [68.0; 89.3] |  | 97.8  [97.0; 99.0] | 0.0% |  |

Abbreviations: CI, confidence interval; ELISA, enzyme-linked immunosorbent assay; *I^2^*, Inconsistency

^a^ Mann-Whitney test
