# Supplementary material for: Diagnostic accuracy of serological tests for the diagnosis of Chikungunya virus infection: A systematic review and meta-analysis
Source: PLoS Negl Trop Dis. 2022 Feb 4;16(2):e0010152. doi: 10.1371/journal.pntd.0010152 (PMC8849447; doi:10.1371/journal.pntd.0010152)
Supplement: Attachment — Submitted filename: Response to reviewers.doc [file pntd.0010152.s011.doc]

Dear Editor,

We are grateful for allowing us to resubmit a significantly revised version of the manuscript to this journal. We also thank the reviewers for their positive, constructive, and valuable feedback. We have responded to all the points and queries carefully and have revised the manuscript incorporating all the suggestions to improve the quality of the manuscript (with changes highlighted yellow for easy reference).

Editor:

*Both reviewers raised important concerns about the study. I agree with them that the overall pooled sensitivity analysis is not worthwhile, as it is important to know (and compare) the performance of tests according to their characteristics and uses. Combining tests that detect IgM antibodies, IgG antibodies, neutralizing antibodies and antigen makes no sense as they have different uses and applications. Likewise, the combination of rapid tests (which show poor performance) with ELISA and IFA to obtain an overall sensitivity does not contribute to the understanding of the performance of these diagnostic methodologies. Just as important, it is not worth combining studies that evaluated samples from the acute and convalescent phases of the disease to determine the overall pooled sensitivity.*

Response: We thank the editor for this comment, and we agree with the editor and the two reviewers. We had simplified the overall diagnostic accuracy of the tests. We focus on subgroup analysis of the analytes to better reflect the utility of the tests for chikungunya infection.

Editor:

*I suggest that overall pooled analysis be removed and that the paper focus on the subgroup analysis, comparing test performance according to the type of test (RDT, ELISA, IFA), the type of antibody detected (IgM, IgG, …), and the timing of sampling (acute, convalescent). Such an approach will best answer which antibody (and which test method) should be used in the acute and in the convalescent-phases of the disease. If possible, further assessment of tests sensitivity according to study design and reference standard for comparison will be good.*

Response: We thank the editor for this suggestion. We now focus on the analysis of each analyte (antigen, IgM, and IgG), and the characteristics of each analyte were available in a separate table (Tables 1 to 3). Based on the information available, further evaluation based on test format, in-house versus commercial test and time of sample collection were included.

Further assessment based on study design was available in the quality assessment section. In the previous manuscript, we did the analysis regardless of the analytes. Here, we stratified the analysis based on the analytes (IgM and IgG).

Changes (Line 339): We compared the diagnostic performance of the case-control and cohort/partial cohort partial case-control study design (Table 10). For antigen detection test, analysis based on study design was not done because there is only one study with a case-control study design (Table 1). For the IgM detection test, a significant difference (P=<0.05) was found in the sensitivity and specificity of the two study designs. Meanwhile, for the IgG detection test, a significant difference was found only for the specificity of the two study designs. Overall, the diagnostic accuracy of the case-control study was higher than the cohort/partial cohort partial case-control study.

**Table 10 Subgroup analysis for study design.**

|  | Number of index test | Pooled Sensitivity | | *P*-value | Pooled Specificity | | *P*-value |
| --- | --- | --- | --- | --- | --- | --- | --- |
|  |  | Percentage  [95% CI] | *I*^2^  [95% CI] |  | Percentage  [95% CI] | *I*^2^  [95% CI] |  |
| IgM | | | | | | |  |
| Case-control | 10 | 93.1  [86.3; 96.7] | 72.5%  [47.9; 85.5] | 0.001 ^a^ | 99.3  [98.1; 99.7] | 0.0%  [0.0; 0.0] | <0.001 ^a^ |
| Cohort/partial cohort partial case-control | 38 | 83.2  [62.2; 93.7] | 92.4%  [90.5; 93.9] |  | 96.1  [94.0; 97.5] | 47.4%  [23.0; 64.0] |  |
| IgG | | | | | | |  |
| Case-control | 6 | 95.0  [89.6; 97.7] | 76.3%  [46.8; 89.4] | <0.905 ^a^ | 99.8  [84.1; 100] | 0.0% | 0.015 ^a^ |
| Cohort/partial cohort partial case-control | 9 | 94.3  [82.6; 98.3] | 65.3%  [29.4; 83.0] |  | 94.6  [89.0; 97.4] | 0.0%  [0.0; 58.1] |  |

Abbreviations: CI, confidence interval

^a^ Mann-Whitney test

Further assessment based on reference standard was not done because all the antigen detection test studies used the molecular method as reference standard except for one study that included virus isolation as the reference standard (see Table 1). For IgM and IgG detection tests, it is very difficult to extract the data based on the reference standard. A variation of reference standards was used in a single study, and the lack of raw data presented by the studies caused the difficulty in performing this analysis. A description on this difficulty is now included in the manuscript.

Changes (Line 505): Analysis based on the reference standard used was not done in this study due to the variations of the reference standard even in a single study. Most studies briefly mentioned the reference standard used, and raw data were not provided. Thus, it is difficult to extract data based on the reference standard and perform analysis.

Editor:

*Another concern is the fact that: “Most of the studies did not specify the time of sample collection”. The lack of this information introduces an important bias, making it difficult to properly interpret sensitivity because sampling time is critical to determine the performance of serological tests. As stated above, meta-analysis should be performed for subgroups based on the time of sampling (acute vs. convalescent). Please address these issues so that the manuscript can be considered for publication on PLOS NTD.*

Response: Dear Editor, thanks for raising this concern, and we apologize for the oversight. The information on the time range of sample collection for each analyte is now added to the manuscript. In summary, the range of time of sample collection for antigen detection test was 1 to 20 days (Line 228), IgM detection test was 1 to 40 days (Line 264), and IgG detection test was 7 to 90 days (Line 287). Since acute samples were used for antigen detection test and convalescent samples were used for IgG detection test, further analysis based on sampling time is not performed for these two analytes. We had also added the lack of this information (sampling time) as a limitation in the strengths and limitations.

Changes (Line 525): Second, only a subset of the studies included in this review provides information with respect to the time of sample collection. The information on the sampling time is important as the detection efficiency of the test varies depending on the presence of the analytes in the patient’s sample.

Editor:

We cannot make any decision about publication until we have seen the revised manuscript and your response to the reviewers' comments. Your revised manuscript is also likely to be sent to reviewers for further evaluation.

Response: Thank you, Editor, for giving us the opportunity to resubmit this manuscript.

Reviewer #1:

(Methods)

1. *Objectives are clearly stated, and methods are appropriate for an overall assessment of test accuracy.*

Response: We thank the reviewer for the positive comment.

2. *However, some methodological choices need to be better justified. For example, authors considered high risk of bias if a study excluded borderline or equivocal results from the analysis or used multiple reference tests. However, equivocal results are usually not to be interpreted as either positive or negative, thus assigning these cases as either TP/FP/FN/TN can biased the results. This is of particular importance if studies are assigning these cases differently. To that point, an overall description on how each study classified equivocal results will help understanding this potential source of heterogeneity.*

Response: We thank the reviewer for raising this important point, and we apologize for the oversight. We agreed that equivocal or borderline results should not be interpreted as either positive or negative. Therefore, we re-evaluated each study using QUADAS-2, and the result for risk assessment was revised (Line 351). We also updated the discussion section (Line 486).

Changes (Line 351): Based on the QUADAS-2 tool, nine (24.3%) and six (16.2%) studies had a high risk of bias with regards to the patient selection and index test, respectively (Figs 8 and 9).

Changes (Line 486): For the index test domain, almost all the studies did not mention whether the index test results were interpreted without knowing the result of the reference standard. There is a high risk of bias as the interpretation of index test results can be influenced by knowledge of the reference standard. The ELISA-based test’s results were categorized into positive, borderline (or equivocal), and negative based on the obtained OD (absorbance) or a ratio. To simplify the analysis, some studies coded the borderline or equivocal samples as positive (41) and negative (31, 50). One study coded equivocal results for the immunochromatographic test as negative (31). Although not described in the study, the equivocal result for ICT can be defined as ambiguous test lines observed. Inconclusive results (borderline or equivocal) should be excluded from analysis as it will increase the risk of bias. Nevertheless, the number of borderline and equivocal samples was very small in this study. Thus, the inclusion of these data will not affect the general result of the meta-analysis.

3. *Similarly, for diseases in which no gold standard testing is adequate throughout most of the course of the disease, it is reasonable that a combination of tests is used to assure a correct classification. Authors also mention that only studies that used direct diagnosis methods (antigen detection, viral isolation or PCR) as reference standards were used. This limits the studied population to the cases that sought medical care early enough in the course of disease.*

Response: We thank the reviewer for this comment. Indeed, there is no gold standard testing for chikungunya disease, and thus the combination of tests assures a correct classification. The previous manuscript categorized the reference standard as in-house, commercial, or a combination of tests. We have now listed out the reference standard used in each study in Tables 1 to 4, and we would like to highlight that the direct detection method (antigen detection, viral isolation, or PCR) mentioned in the manuscript (Line 108) is only assigned to the antigen detection test. There is no restriction on the reference standard for antibody detection tests. Further elaboration of these inclusion criteria was included in the manuscript.

Changes (Line 499): This meta-analysis specifies direct detection methods such as virus isolation and molecular-based method as the reference standard for antigen detection tests to ensure that the samples were collected during the viral stage.

4. *Authors also opted to combine acute- and convalescent-phase data (when available) for an overall description of IgM and IgG testing accuracy. Although not wrong, these tests are not intended for acute-phase testing, thus the overall accuracy combining both phases has limited applicability.*

Response: We thank the reviewer for raising this important point. The previous manuscript combined acute and convalescent data for few studies on IgM detection tests. Since we have separated the data based on analyte, we did not pool acute and convalescent data. Analysis based on time of sample collection was only done for the IgM detection test, and the acute and convalescent data were identified and coded with superscript (^a^ and ^b^) in Table 2.

Changes (Line 265): For studies that provide complete data for the day of sample collection, we categorized the time of sample collection into acute (≤ 7 days post symptom onset) and convalescent (>7 days post symptom onset) (Table 2).

Reviewer #2:

(Methods)

*This is a well written manuscript that is of particular interest to diagnosticians of tropical infections.*

Response: We are very grateful to the reviewer for this positive comment.

Reviewer #1:

(Results)

1. *Results are clearly presented although additional information might be useful to interpret the analysis.*

Response: We thank the reviewer for this positive feedback.

2. *In Table 1, it would be important to know which clinical setting the studies took place (outpatient, hospitalized, etc).*

Response: We thank the reviewer for this suggestion. Most of the studies use retrospective samples. These samples have been analysed and confirmed by any diagnostic tests in a previous outbreak or study. Rarely, the information on the clinical settings is provided in the diagnostic accuracy study. Furthermore, chikungunya is a self-limiting disease and hospitalisation is not frequently needed.

3. *It would also be useful to know which type of reference test was used (antigen detection, viral isolation, PCR).*

Response: We thank the reviewer for this comment. We have listed the reference standard used in each study in Tables 1 to 4 and added the description of the reference tests used for each analyte in the manuscript. The reference test used was not limited to antigen detection, viral isolation, and PCR.

Changes (Line 227): All seven antigen detection studies use either molecular method and/or virus isolation as a reference test, and none of the antigen detection tests is commercially available.

Changes (Line 242): A variety of reference tests were used in the diagnostic accuracy studies of the IgM detection test. These include molecular methods, in-house serology tests and commercial kits (Table 2). Some studies use the molecular method to confirm CHIKV infection for the samples collected on the first day of symptom appear, and subsequent samples were collected from the same patient, which was used for IgM detection test.

Changes (Line 286): The reference standards used for IgG detection test study include commercial kit, in-house ELISA, IFA or PRNT.

4. *The average and range of days of symptoms of the tested sample will also be important for interpretation of Table 3 summarizing IgM/IgG sensitivity.*

Response: We thank the reviewer for this suggestion. The information on the time range of sample collection for each analyte is now added to the manuscript. In summary, the range of time of sample collection for antigen detection test was 1 to 20 days (Line 228), IgM detection test was 1 to 40 days (Line 264), and IgG detection test was 7 to 90 days (Line 287).

Analysis based on time of sample collection was only done for IgM detection test because of the wide range of time of sample collection. We have added a description explaining this analysis in the manuscript.

Changes (Line 390): In this meta-analysis, the time of sample collection for IgM detection tests range from day 1 to day 40 post symptom onset. This wide range of time of samples collection is theoretically acceptable as IgM antibodies are known to appear as early as days 3 to 4 from the onset of illness and can persist up to 3 months (57). However, our meta-analysis revealed that the sensitivity of the IgM detection test for samples collected in the acute phase was very low (26.2%) compared to samples collected in the convalescent phase of the infection (98.4%). This result is in coherence with the review by Natrajan et al. (58), where IgM tests achieve a 100% detection rate for CHIKV for samples collected more than 6 days of symptom onset. In short, although IgM antibodies start to develop from day 2 onwards of CHIKV infection, the level can be too low in the initial phase and cannot be detected by most serological tests. Therefore, an IgM detection test is not recommended for samples collected in the acute phase of infection.

5. *Similarly, in the subgroup analysis of commercial tests it might be also important to summarize the type of study and average days of symptom of samples used for these calculations (at a quick glance it seems like most are partial cohort/case-control for most companies and cohort for most SD).*

Response: We thank the reviewer for highlighting this point. The data for subgroup analyses of commercial tests were made available in supporting information Table S1, S2 and S3. In addition, the description of the type of study and the time range of sample collection was added to the manuscript.

Changes (Line 316): A meta-analysis was performed for nine commercial tests detecting IgM and IgG antibodies (Table 9). The data for meta-analysis based on test format were available in supplementary tables S1, S2, and S3. Data for samples collected <7 days post symptom onset were excluded from the analysis since most manufacturers recommended using samples collected in the range of 6 to 8 days of symptom onset. Mainly, cohort or partial cohort partial case-control study designs were used for the commercial kit studies. Only two studies use case-control study design (39, 41).

6. *For Table 7, it is unclear what number of index tests mean. Is it the number of studies evaluating the test? Euroimmun only has one CHIKV ELISA IgM commercial test, for example, hence my confusion.*

Response: We wish to thank the reviewer for pointing this out. The word “index test” has now been amended to “number of studies” (Table 9).

Reviewer #2:

(Results)

*The results are well presented as are the figures.*

Response: We are very grateful to the reviewer for this positive comment.

Reviewer #1:

(Conclusions)

1. Conclusions are supported by the data presented.

Response: We are very grateful to the reviewer for this positive comment.

2. Some important limitations were not discussed. Authors state that most of the studies did not specify the time of sample collection. This can be an issue since the reference tests they selected for are all direct methods. If IgM/IgG testing is being done in the same sample as the reference test, this can artificially decrease sensitivity because it is being used at an inappropriate timing. This is likely one of the reasons for the heterogeneity found.

Response: First of all, we would like to highlight that the direct detection method (antigen detection, viral isolation, or PCR) is only assigned to the antigen detection test (Line 108). Thus, there should be no issue with the inappropriate timing of sample collection for IgM and IgG detection tests.

We had also added the lack of sampling time information as a limitation in the manuscript.

Changes (Line 525): Second, only a subset of the studies included in this review provides information with respect to the time of sample collection. The information on the sampling time is important as the detection efficiency of the test varies depending on the presence of the analytes in the patient’s sample.

3. Similarly, another source of heterogeneity not discussed it the intentional pooling of acute- and convalescent-phase samples for the overall sensitivity calculations, particularly for IgM/IgG. In the subgroup analysis, there is likely a combination of factors that were only assessed individually that potentially justifies the heterogeneity (e.g. acute- vs convalescent-phase samples, study type, etc.).

Response: We thank the reviewer for this comment. Since we have separated the data based on each analyte, we did not pool acute and convalescent data.

4. Additionally, although generally the statement “Detection of IgG alone indicates past infection, while IgM positive with or without IgG indicates recent infection” is correct, I would point to caution as detection of IgG alone might not necessarily indicate past infection if a false negative in the IgM testing occurs.

Response: We thank the reviewer for highlighting this. We had removed the statement and amended with “In summary, IgM and IgG antibody detection tests need to be carried out simultaneously for a more accurate diagnosis of CHIKV infection in the convalescent phase of the infection.” (Line 435)

Reviewer #2:

(Conclusions)

1. *The authors have missed some of the main limitations especially sample timing, reference comparators and sample composition. They are mentioned but only in passing - more emphasis needs to be placed on these important potential bias factors.*

Response: We thank the reviewer for this comment. We have now added limitations and descriptions on sample timing and quality of reporting in the manuscript.

Changes (Line 525): Second, only a subset of the studies included in this review provides information with respect to the time of sample collection. The information on the sampling time is important as the detection efficiency of the test varies depending on the presence of the analytes in the patient’s sample. Other information such as blinding the reference test result when interpreting index test, the expertise of the person who performs IFA, and samples conditions is also important so that sources of variation can be appreciated, and applicability can be evaluated. We strongly recommend employing a prospective cohort study design and a full report on the methodology associated with reference and index tests for a more accurate estimation of the diagnostic accuracy for CHIKV serological testing.

2. *I am concerned about the lack of detail in the timing of the sample (days of illness) information provided in this meta-analysis.*

Response: We thank the reviewer for this concern. The information on the range of time of sample collection for each analyte is now added in the manuscript. In summary, the range of time of sample collection for antigen detection test was 1 to 20 days (Line 228), IgM detection test was 1 to 40 days (Line 264), and IgG detection test was 7 to 90 days (Line 287).

3. *While the authors state that this was a problem in the abstract it is not mentioned as a limitation of the study.*

Response: We thank the reviewer for this comment, and we had added the lack of this information (sampling time) as a limitation in the section strengths and limitations.

Changes (Line 525): Second, only a subset of the studies included in this review provides information with respect to the time of sample collection. The information on the sampling time is important as the detection efficiency of the test varies depending on the presence of the analytes in the patient’s sample.

4. *It is noted that this information is included in table 5 of the study and illustrates the point well regarding sample timing especially in the acute phase of the study. The authors have overlooked the importance of this observation as it is imperative that the days of illness be included in any diagnostic accuracy study because if there is a large number of acute phase samples included in the study then it is unlikely that there will be detectable levels of antibodies available and therefore this will be reflected in the results indicating low sensitivity as illustrated in the last rows of table 5. This conclusion is fine because you cannot detect something that is not present. Conversely, it is simple to make any insensitive test look good by including large numbers of convalescent samples in the study group. It is strongly recommended that additional comment or information be provided in the Discussion section regarding the impact of the timing of the sample in the context of days of illness presentation.*

Response: We thank the reviewer for this comment and recommendation. We have added the information on the range of time of sample collection for each analyte, and we found that acute samples (1 to 20 days post symptom onset) were used for antigen detection test and convalescent samples (7 to 90 days post symptom onset) were used for IgG detection test. Thus, further analysis based on the time of sampling is not performed for these two analytes. There will be no issue with the inappropriate use of only acute samples for the analytes analysed in this study.

Analysis based on time of sample collection was only done for IgM detection test because of the wide range of time of sample collection (1 to 40 days post symptom onset). We have added a description explaining this analysis in the manuscript.

Changes (Line 390): In this meta-analysis, the time of sample collection for IgM detection tests range from day 1 to day 40 post symptom onset. This wide range of time of samples collection is theoretically acceptable as IgM antibodies are known to appear as early as days 3 to 4 from the onset of illness and can persist up to 3 months (57). However, our meta-analysis revealed that the sensitivity of the IgM detection test for samples collected in the acute phase was very low (26.2%) compared to samples collected in the convalescent phase of the infection (98.4%). This result is in coherence with the review by Natrajan et al. (58), where IgM tests achieve a 100% detection rate for CHIKV for samples collected more than 6 days of symptom onset. In short, although IgM antibodies start to develop from day 2 onwards of CHIKV infection, the level can be too low in the initial phase and cannot be detected by most serological tests. Therefore, an IgM detection test is not recommended for samples collected in the acute phase of infection.

5. *There is little or no mention of the influence of Chikungunya prevalence on the outcome of diagnostic accuracy studies. While there is mention of this in the section on bias the implications are not really clear to the reader (i.e., high vs low prevalence in prospective study design and high versus low n positives in a case-control design) Please provide additional information regarding the implications of such bias in the sample composition especially in case control studies.*

Response: We thank the reviewer for highlighting this. The sensitivity and specificity of a test often vary with disease prevalence. We conducted a t-test and found that the prevalence of chikungunya disease in establishing the accuracy of the test was similar for cohort (46.5%) and case-control study designs (55.98%) (p-value = 0.1544). Thus, we think that the sample composition in the case-control study design will not affect the heterogeneity found in this meta-analysis.

6. *There is an absence of information regarding the influence of the reference comparator in the diagnostic accuracy study. This is an especially important source of bias often an inappropriate reference comparator is selected or an inappropriate diagnostic cutoff is used which can significantly bias the results. Furthermore, it is often more appropriate that a final patient case results (true disease status) of “Chikungunya positive” or “Chikungunya negative” be used for calculating the sens and spec because the true disease status is more relevant that comparing positivity scores against an randomly assigned diagnostic cutoff.*

Response: We thank the reviewer for this comment. Since there is no gold standard for diagnosing CHIKV infection, we rated all the reference standards as low risk of bias. However, we did not deny that the variation in the reference standard used would impact the test’s accuracy. Analysis based on reference standard was not perform due to high variations in the reference standard used even in a single study. Some use more than one reference standard and most of the time, raw data were not provided. We had updated the discussion on reference standard in the manuscript.

Changes (Line 497): There was no bias recorded for the reference standard domain. Different reference standards were used in the diagnostic accuracy studies since no gold standard is available for diagnosing CHIKV. This meta-analysis specifies direct detection methods such as virus isolation and molecular-based method as the reference standard for antigen detection tests to ensure that the samples were collected during the viral stage. Some studies use molecular tests as the reference standard (36, 41), and subsequent samples collected from the same patient were used for IgM or IgG accuracy study. Although not wrong, the immune response of each patient can be varied against CHIKV. Some patients may not develop antibodies against CHIKV, and thus the use of these samples will lead to the low sensitivity of the test. Analysis based on the reference standard used was not done in this study due to the variations of the reference standard even in a single study. Most studies briefly mentioned the reference standard used, and raw data were not provided. Thus, it is difficult to extract data based on the reference standard and perform analysis.

7. *Line 420. The authors have stated the following “ Second, we received no response from the corresponding authors (24, 32) regarding discrepancies in the raw data and diagnostic accuracy.” It is not clear what the authors mean by this statement as there is no additional information in the manuscript regarding these two papers. It would appear that because these two studies departed from the main theme of generally high diagnostic accuracy that there is a methodological problem with the study. The study of Huits et al describes the Arkay Chikungunya antigen ICT which described “fair diagnostic sensitivity for ECSA genotype chikungunya, but low sensitivity for Asian genotype, and poor overall specificity.”The study of Blacksell et al describes a diagnostic accuracy study evaluating the Standard Diagnostics IgM ELISA using a series of acute-phase samples compared to a gold standard reference comparators with the conclusion that the essay had low sensitivity which is a reflection on the days of illness of the samples. In fact, figure 4, indicates that for that particular study there was Low risk of bias or applicability concerns. Because of the lack of detail, I recommend that this sentence be removed from the manuscript as the implications are unfair to the authors.*

Response: We thank the reviewer for this comment. We agreed with the suggestion. The discrepancy was related to the raw data and the sensitivity and specificity presented in their articles. We agreed to remove this statement since the discrepancy did not affect the overall diagnostic accuracy reported in this study.

Reviewer #1:

(Editorial and Data Presentation Modification)

1. *The number of CHIKV serological tests increase tremendously after the Indian Ocean outbreaks in 2004 - As in number of tests used or different tests commercially available?*

Response: We thank the reviewer for this query. We have now amended this sentence to “The number of studies on CHIKV serological tests increases tremendously after the Indian Ocean outbreaks in 2004”. (Line 92)

2. *Line 126: We included only the optimized index test data in the analysis for studies developing serological tests using a different batch of antigens or antibodies.- What is optimized index test data?*

Response: We thank the reviewer for this query. The optimized index test data refers to the highest diagnostic accuracy of the index test. We have now amended this sentence to “For studies developing serological tests either with different antigens or antibodies of the same test format, only the optimized index test data (highest diagnostic accuracy) were extracted for analysis.” (Line 133)

3. *Line 169: For commercial tests (specific brand) with two or more reports, meta-analyses were done to determine their diagnostic accuracy. We included only samples collected after 7 days from the onset of clinical symptoms for this analysis.- Clarify that for 2+ studies describing accuracy of a commercially available test, a meta-analysis per manufacturer/brand was done. This sentence was not clear to me until I read the results.*

Response: We thank the reviewer for highlighting this. We have now amended this sentence to “For commercial tests (specific brand) with two or more diagnostic accuracy studies, meta-analyses were done according to the individual commercial kit” (Line 176).

4. *Clarification on the use of “acceptable reference standards” (line 347) is needed. Are the authors suggesting the reference standards they used (PCR, viral isolation or antigen detection) are not acceptable?*

Response: We thank the reviewer for highlighting this. We have now removed the sentence and replace it with “We strongly recommend employing a prospective cohort study design and a full report on the methodology associated with reference and index tests for a more accurate estimation of the diagnostic accuracy for CHIKV serological testing.” (Line 531)

Reviewer #2:

(Editorial and Data Presentation Modification)

*No comments*

Reviewer #1:

(Summary and General comments)

1. *The overall accuracy analysis seems to have limited applicability due to the compilation of studies with such different methodologies (e.g., grouping different detection methods – ELISA, RDT, IF; sample type, etc.). In that sense, the subgroup analysis is more informative and perhaps the most important aspect of this work.*

Response: We thank the reviewer for this comment, and we agreed to it. We now focus on the analysis of each analyte (antigen, IgM, and IgG), and the characteristics of each analyte were available in a separate table (Table 1 to 3). Based on the available information, further evaluation based on test format, in-house vs commercial test, and time of sample collection were included.

2. *The risk of bias assessment needs better theoretical background justifying authors choices in qualifying the information (i.e., why were studies that used a combination of reference tests classified as having higher risk of bias?). Perhaps the novelty factor of a meta-analysis lies on the discussion of the heterogeneity of the data. As described, many aspects of this discussion were lacking.*

Response: We thank the reviewer for highlighting this, and we apologize for the oversight. We re-evaluate the risk of bias using QUADAS-2 according to the information provided by the reviewer. More information was added for the discussion on the study design and risk of bias.

Changes (Line 497): There was no bias recorded for the reference standard domain. Different reference standards were used in the diagnostic accuracy studies since no gold standard is available for diagnosing CHIKV. This meta-analysis specifies direct detection methods such as virus isolation and molecular-based method as the reference standard for antigen detection tests to ensure that the samples were collected during the viral stage. Some studies use molecular tests as the reference standard (36, 41), and subsequent samples collected from the same patient were used for IgM or IgG accuracy study. Although not wrong, the immune response of each patient can be varied against CHIKV. Some patients may not develop antibodies against CHIKV, and thus the use of these samples will lead to the low sensitivity of the test. Analysis based on the reference standard used was not done in this study due to the variations of the reference standard even in a single study. Most studies briefly mentioned the reference standard used, and raw data were not provided. Thus, it is difficult to extract data based on the reference standard and perform analysis.

Reviewer #2:

(Summary and General comments)

*The authors have missed some of the main limitations especially sample timing, reference comparators and sample composition. They are mentioned but only in passing - more emphasis needs to be placed on these important potential bias factors to provide a balanced viewpoint.*

Response: We thank the reviewer for this comment. We have now added limitations and descriptions on sample timing and quality of reporting in the manuscript.

Changes (Line 525): Second, only a subset of the studies included in this review provides information with respect to the time of sample collection. The information on the sampling time is important as the detection efficiency of the test varies depending on the presence of the analytes in the patient’s sample. Other information such as blinding the reference test result when interpreting index test, the expertise of the person who performs IFA, and samples conditions is also important so that sources of variation can be appreciated, and applicability can be evaluated. We strongly recommend employing a prospective cohort study design and a full report on the methodology associated with reference and index tests for a more accurate estimation of the diagnostic accuracy for CHIKV serological testing.
