# Supplementary material for: Diagnostic accuracy of serological tests for the diagnosis of Chikungunya virus infection: A systematic review and meta-analysis
Source: PLoS Negl Trop Dis. 2022 Feb 4;16(2):e0010152. doi: 10.1371/journal.pntd.0010152 (PMC8849447; doi:10.1371/journal.pntd.0010152)
Supplement: Attachment — Submitted filename: Response to reviewers.docx [file pntd.0010152.s012.docx]

Dear Editor,

Thank you for allowing us to resubmit our manuscript "Diagnostic accuracy of serological tests for the diagnosis of Chikungunya virus infection: a systematic review and meta-analysis" to PLOS Neglected Tropical Diseases and the consideration to accept this manuscript. We appreciate the comments and suggestions from the reviewers. We have revised the manuscript incorporating all the suggestions, and the changes made were highlighted in green for easy reference. Yellow highlighted text indicates revised grammar and phrasing.


**Methods**


Reviewer #1: Objectives are clearly stated, and methods are appropriate. I appreciate that the authors justified some of their methodological choices so the readers can easily understand the rationale some assessments are based. Authors also justified that there was a low proportion of equivocal results, so the potential impact in the analysis is minimal (although there was not a clear statement of what that proportion was).

Response: We thank the reviewer for the positive comments. Regarding the low proportion of the equivocal results, only 19 samples (from a total of 10563) coded borderline or equivocal samples as either positive or negative. We have now added the information in the manuscript (Line 505).

Changes (Line 505): The inclusion of inconclusive results (borderline or equivocal) in the analysis will increase the risk of bias. However, as the number of borderline and equivocal samples (19 out of 10563) in this study was very small, the inclusion of these data will not affect the general result of the meta-analysis.

Reviewer #2: The revisions made by the authors are acceptable.

Response: Thank you, reviewer.


**Results**

Reviewer #1: Results are clearly presented although some figures might be combined for a more concise display of the information.

Response: Thank you, reviewer, for the positive comment and suggestion. In our humble opinion, combining the figures will conceal the important information that we would like to highlight. Therefore, we would like to retain all the figures.

It would still be useful to have a description of both the clinical setting the studies took place (outpatient, hospitalized, etc) and geographic location of where the studies took place to have an idea of the epidemiological scenario in which it took place. A brief text summary (e.g., most (XX%) of studies occurred with outpatient patients retrospectively and XX% took place in endemic regions) would suffice. That gives the reader context to what these studies represent.

Response: Thank you, reviewer, for the suggestion. We have now added a brief description of the study participants as below.

Changes (Line 202): Most of the studies (70%) did not specify the time of sample collection and the clinical background of the study participants. Only five studies (14.3%) specified that the samples were collected from hospitalised patients, and six studies (17.1%) used patient samples collected during CHIKV outbreaks.

Reviewer #2: The revisions made by the authors are acceptable.

Response: Thank you, reviewer.

**Conclusions**


Reviewer #1: Conclusions are supported by the data presented although some phrasing needs to be revised. Some limitations were discussed as the results were presented (IgG RDT having higher accuracy) but need to be reinforced in the discussion as well.

Response: We thank the reviewer for the positive comment and suggestion. The discussion on the IgG rapid test having higher accuracy was available in line 442 in the manuscript.

Line 442: Among these tests, rapid tests are attractive because they are easy to perform, do not require expensive equipment, and the result can be obtained within a minute. Two commercial IgG rapid tests with promising diagnostic accuracy are recently available (38, 47), but further evaluation with multiple prospective cohort studies is needed to provide comprehensive data for meta-analysis.

Although IgG RDT had the highest accuracy (Table 8), it is not reinforced in the conclusion because the different test formats (ELISA, IFA and rapid test) showed no difference in their diagnostic performance (p-value = >0.05). We have now rephrased and revised the conclusion (Line 555).

Changes (Line 555): According to our meta-analysis, depending on the time of samples collection, antigen and antibody-based serological tests can accurately diagnose CHIKV. Antigen detection tests are an effective diagnostic test for samples obtained during the acute phase (1 to 7 days post symptom onset), whereas IgM and IgG detection tests can be used for samples collected in the convalescent phase (> 7 days post symptom onset). In correlation to the clinical presentation of the patients, the combination of the IgM and IgG tests can differentiate recent and past infections. Several commercial IgM and IgG assays have been recognised as promising, which included kits from Euroimmun (Lübeck, Germany), Abcam (Cambridge, UK), and Inbios (Seattle, WA). The caveats to the finding of this meta-analysis are inconclusive reporting of data in this review and low quality of reporting in diagnostic test accuracy studies.

Additionally, although generally the statement “Detection of IgG alone indicates past infection, while IgM positive with or without IgG indicates recent infection” is correct, I would point to caution as detection of IgG alone might not necessarily indicate past infection if a false negative in the IgM testing occurs.

Response: Thank you, reviewer. The statement “Detection of IgG alone indicates past infection, while IgM positive with or without IgG indicates recent infection” has been removed in the previously revised manuscript.

Lastly, the authors mention throughout the discussion/conclusion that IgM and IgG testing of the convalescent-phase sample is recommended to differentiate between recent and past infections. I think the authors might mean testing IgM/IgG in the acute-phase sample to help differentiate between recent and past infections, please revise. Although IgM sensitivity is low for acute-phase samples, testing only convalescent-phase samples would not differentiate between recent or past infection since the patient would have had enough time to develop antibodies even in a primary infection.

Response: Thank you, reviewer, for this comment. First of all, we have added the definition of the acute (i.e., 1 to 7 days post symptom onset) and convalescent phase (i.e., >7 days post symptom onset) of CHIKV infection (Line 371). Second, we strongly suggest the IgM and IgG testing of the convalescent-phase samples for the following reasons:

1. In our discussion (Line 407), we mentioned, “while IgM antibodies begin to develop from day 2 of CHIKV infection, the level can be way below the detection limit of most serological assays. Thus, the IgM detection test is not recommended for samples taken during the acute phase of infection”. Suggesting testing of IgM for acute-phase samples will increase the false-negative results.
2. In the WHO guideline, detecting IgM antibodies in a single serum collected in the acute or convalescent phase is defined as a recent infection (Line 413). Therefore, the presence of IgM antibodies in the convalescent phase (after 7 days of symptom onset) could indicate a recent infection.
3. We agreed that detection of IgG in the convalescent-phase samples would not differentiate recent and past infections. We have added the need for a second serum to rule out past infection (Line 435). We had also added that obtaining second samples from the patients is not always possible. In such situation, the presence of the CHIKV IgG antibody in a single sample should be interpreted in correlation with the clinical presentation of the patients.

Changes (Line 371): During CHIKV infection, once the virus enters the host, it replicates and causes viremia, which lasts about 7 days. The patient's clinical manifestations, such as fever, are closely related to the high viral load during this period (56, 57). The appearance of the antibodies in the following phase is linked to a decrease in viremia. In this meta-analysis, the acute phase is defined as days 1 to 7 following the beginning of symptoms, while the convalescent phase is defined as after 7 days of symptom onset.

Changes (Line 413): According to WHO guidelines, a confirmed CHIKV case is defined as the presence of CHIKV IgM antibodies in a single serum sample taken during the acute or convalescent phases, indicating recent infection (10).

Changes (Line 435): As CHIKV IgG antibodies persist for years, a second sample should be collected three weeks apart to rule out past infection. As stated in the WHO guidelines, a recent CHIKV diagnosis can be confirmed if there is a fourfold increase in IgG titer between the samples (10). However, obtaining second samples from the patients is not always possible. In such situation, the presence of the CHIKV IgG antibody in a single sample should be interpreted in correlation with the clinical presentation of the patients.

Changes (Line 446): In summary, IgM and IgG antibody detection tests had high accuracy (>90%) for samples collected in the convalescent phase of CHIKV infection. The detection of IgM indicates recent infection, while a second sample collected at least 3 weeks apart is needed for the positive IgG test to rule out past infection.

Reviewer #2: The revisions made by the authors are acceptable.

Response: Thank you, reviewer.


**Editorial and Data Presentation Modifications?**

Reviewer #1: Figs 2-7 seems to be somewhat redundant with Figure S1.
- The stratification is relevant, but in interest of efficiency I would suggest combining them into maybe Figure S1 with three additional columns to denote the test type (RDT, ELISA), whether or not it is a commercial test, and days of symptoms (> or < 7)

Response: Thank you, reviewer, for the comment and suggestion. Figure S1 is a forest plot based on different analytes (antigen and antibodies), which provide limited information. Thus, it was included in supporting information. In our humble opinion, adding three additional columns in one large figure will conceal the important aspect of the information that we would like to highlight. Therefore, we would like to retain the figures.

Ag detection test: no difference between rapid test and elisa
- Very large confidence for Sp heterogeneity, I would like to have the authors discuss possible explanations in the discussion

Response: Thank you, reviewer, for pointing this out. The large confidence interval for the specificity heterogeneity was due to the low sample size. We have added this in the limitation, and a brief discussion was included in the manuscript.

Changes (Line 539): One of the characteristics that affect the width of the confidence interval is the sample size (63). Due to the small sample size, some of the analyses showed a wide range of 95% confidence interval. For example, the heterogeneity (I2) for the specificity of the rapid (95%CI 0 to 85.7) and ELISA-based (95%CI 0 to 85.1) antigen detection tests had a wide 95% confidence interval. A similar observation was seen for the specificity of the rapid IgG test (95% CI 0.0 to 100). Wide confidence often indicates that the estimated results provide less certain information. Therefore, at this point, we have a low level of certainty for analysis with a wide confidence interval.

- Include I² in the legend for Table 6

Response: We thank the reviewer for pointing this out. We have now included the legend in all the tables.

Line 251: In addition, the sensitivity of the rapid tests was statistically different from ELISA-based (93.4%; 95% CI 81.7 to 97.8; P=0.002) and IFA (99.3%; 95% CI 69.4 to 100; P=0.027)
- I would suggest adding RDTs Se in the phrasing as well for easy reading “In addition, the sensitivity of the rapid tests (42.3%, 95%CI…) …”

Response: Thank you, reviewer, for this suggestion. We have amended the sentence.

Changes (Line 256): The sensitivity of the rapid tests (42.3%; 95% CI 19.2 to 69.4) was statistically different from ELISA-based (93.4%; 95% CI 81.7 to 97.8; P=0.002) and IFA (99.3%; 95% CI 69.4 to 100; P=0.027), while no significant difference was found in the sensitivity of IFA and ELISA-based tests (P=0.414).

Line 267: The forest plot (Fig 5) shows that the sensitivity for samples collected ≤7 days of symptoms onset mostly lies on the left side of the plot. Incoherence with this observation, our meta-analysis shows that the sensitivity of the acute samples was significantly lower than convalescent samples (Table 7).
- It doesn’t seem incoherent. Fig 5 shows most sensitivities within the ≤7 days studies around 0-60% (left skewed) and most sensitivities within >7 days studies around 80-100% (right skewed), meaning lower sensitivity in acute-phase samples.

Response: Thank you, reviewer, for this comment. Indeed, lower sensitivity was observed for the acute-phase samples in Fig 5 and the analysis in Table 7. We have now amended the sentence (Line 273).

Changes (Line 273): The forest plot (Fig 5) shows that the sensitivity estimates for samples collected ≤ 7 days of symptoms onset mostly lies on the left side of the plot. Consistent with this observation, our meta-analysis shows that the sensitivity for the samples collected ≤ 7 days of symptoms onset was significantly lower than samples collected >7 days post symptom onset (Table 7). These results indicate that the IgM detection test had low accuracy for acute-phase samples.

Table S1: Clarify if CHIKjj Detect MAC-ELISA is IgM or IgG (both are available commercially), same for Anti-Chikungunya Virus, Abcam. Similarly for Table 9.

Response: We thank the reviewer for this suggestion. For the CHIKjj Detect MAC-ELISA, MAC denotes IgM antibody capture, which is understandable as IgM assay. For the Anti-Chikungunya Virus, Abcam in Table S1, we have rectified the name.

Changes (Table S1): Anti-Chikungunya Virus, Abcam, UK is changed to Anti-Chikungunya Virus IgM Human ELISA Kit, UK.

Figure 9 could be submitted as a supplementary figure
Response: Thank you, reviewer, and we have moved this figure (Fig 9) to the supporting information (Fig S2).

Line 426: “As IgG antibodies generally persist for years, qualitative detection of both CHIKV IgG and IgM antibodies in the convalescent phase can help to differentiate recent and past infections.”
- I think the authors might mean testing IgM/IgG in the acute-phase sample to help differentiate between recent and past infections, please revise. Although IgM sensitivity is low for acute-phase samples, testing only convalescent-phase samples would not differentiate between recent or past infection since the patient would have had enough time to develop antibodies even in a primary infection.

Response: Thank you, reviewer, for this comment. First, we have added a description to clarify the definition of the acute and convalescent phase of CHIKV infection in the manuscript (Line 371). As mentioned in the previous response, our meta-analysis showed that the IgM detection test had low accuracy for acute-phase samples. Therefore, we suggest an IgM detection test for convalescent-phase samples.

Changes (Line 371): During CHIKV infection, once the virus enters the host, it replicates and causes viremia, which lasts about 7 days. The patient's clinical manifestations, such as fever, are closely related to the high viral load during this period (56, 57). The appearance of the antibodies in the following phase is linked to a decrease in viremia. In this meta-analysis, the acute phase is defined as days 1 to 7 following the beginning of symptoms, while the convalescent phase is defined as after 7 days of symptom onset.

Line 426: “As IgG antibodies generally persist for years, qualitative detection of both CHIKV IgG and IgM antibodies in the convalescent phase can help to differentiate recent and past infections.”

Response: We have now amended the sentence to: “As CHIKV IgG antibodies persist for years, a second sample should be collected three weeks apart to rule out past infection. As stated in the WHO guidelines, a recent CHIKV diagnosis can be confirmed if there is a fourfold increase in IgG titer between the samples (10). However, obtaining second samples from the patients is not always possible. In such situation, the presence of the CHIKV IgG antibody in a single sample should be interpreted in correlation with the clinical presentation of the patients..” Line 435

- Same comment for line 435: “In summary, IgM and IgG antibody detection tests need to be carried out simultaneously for a more accurate diagnosis of CHIKV infection in the convalescent phase of the infection.”

Changes (Line 446): In summary, IgM and IgG antibody detection tests had high accuracy (>90%) for samples collected in the convalescent phase of CHIKV infection. The detection of IgM indicates recent infection, while a second sample collected at least 3 weeks apart is needed for the positive IgG test to rule out past infection.

- And Line 537: “In contrast, IgM and IgG tests can be used for samples collected in the convalescent phase, whereby the combination of the tests can differentiate recent and past infections.”

Changes (Line 559): In correlation to the clinical presentation of the patients, the combination of the IgM and IgG tests can differentiate recent and past infections.

Line 434: “Rapid tests showed the highest diagnostic accuracy among test formats available to detect
CHIKV IgG antibodies, followed by IFA and ELISA-based tests”
- A follow up phrase pointing that there was no statistical difference between RDT, ELISA and IFA sensitivities and that the confidence intervals for the RDT Se and Sp are very large (Table 8) is needed here.

Response: Thank you, reviewer, for this comment. We have now changed the sentence “Rapid tests showed the highest diagnostic accuracy among test formats available to detect CHIKV IgG antibodies, followed by IFA and ELISA-based tests” to “There was no difference in the diagnostic performance of the IgG rapid test, IFA, and ELISA-based test.” (Line 441). We have now added a description of the large confidence intervals for the specificity of RDT in the limitation (Line 542).

Changes (Line 441): There was no difference in the diagnostic performance of the IgG rapid test, IFA, and ELISA-based test. Among these tests, rapid tests are attractive because they are easy to perform, do not require expensive equipment, and the result can be obtained within a minute.

Changes (Line 542): A similar observation was seen for the specificity of the rapid IgG test (95% CI 0.0 to 100).

Reviewer #2: The revisions made by the authors are acceptable however the manuscript still requires some editing for English grammar and phrasing.

Response: Thank you, reviewer, for the positive comments. One of our English native speaker researchers had helped review the manuscript, and we acknowledged her contribution in the acknowledgement.

The corrected grammar and phrasing in the manuscript are highlighted in yellow.

**Summary and General Comments**


Reviewer #1: The manuscript improved significantly with changes in the methodology and text and my main concerns were addressed. Few revisions are still necessary to assure some phrasings are not misinterpreted.

Response: We appreciate the positive comments and suggestions to improve the manuscript. We have revised the manuscript incorporating the constructive suggestion from the reviewers.

Reviewer #2: The revisions made by the authors are acceptable.

Response: Thank you.
